# No effect of testosterone or sexual ornamentation on telomere dynamics: A case study and meta‐analyses

**DOI:** 10.1002/ece3.11088

**Published:** 2024-03-01

**Authors:** Gregory T. Taylor, Alexandra McQueen, Justin R. Eastwood, Andréaz Dupoué, Bob B. M. Wong, Simon Verhulst, Anne Peters

**Affiliations:** ^1^ School of Biological Sciences Monash University Clayton Victoria Australia; ^2^ Groningen Institute for Evolutionary Life Sciences University of Groningen Groningen The Netherlands; ^3^ Present address: Centre for Integrative Ecology Deakin University Burwood Victoria Australia; ^4^ Present address: CNRS Sorbonne Université, UMR 7618, iEES Paris Université Pierre et Marie Curie Paris France

**Keywords:** condition dependence, honesty, life‐history, sexual selection, superb fairy‐wren, trade‐offs

## Abstract

Life‐history theory predicts that reproductive investments are traded‐off against self‐maintenance. Telomeres, the protective caps on the ends of chromosomes, offer a promising avenue for assessing life‐history trade‐offs, as they shorten in response to stressors and are predictive of the remaining lifespan. In males, testosterone frequently mediates life‐history trade‐offs, in part, through its effects on sexual ornamentation, which is an important aspect of reproductive investment. However, studies of within‐individual associations between telomere dynamics and sexual ornamentation are limited in number and have produced mixed results. Furthermore, most such studies have been observational, making it difficult to discern the nature of any causal relationship. To address this, we used short‐acting testosterone implants in free‐living male superb fairy‐wrens (*Malurus cyaneus*) to stimulate the production of a sexual ornament: early moult into a costly blue breeding plumage. We found no evidence that elevated testosterone, and the consequent earlier moult into breeding plumage, accelerated telomere shortening. We therefore followed up with a systematic review and two meta‐analyses (28 studies, 54 effect sizes) exploring the associations between telomeres and (1) testosterone and (2) sexual ornamentation. In line with our experimental findings, neither meta‐analysis showed an overall correlation of testosterone or sexual ornamentation with telomere length or telomere dynamics. However, meta‐regression showed that experimental, compared to observational, studies reported greater evidence of trade‐offs. Our meta‐analyses highlight the need for further experimental studies to better understand potential responses of telomere length or telomere dynamics to testosterone or sexual ornamentation.

## Graphical abstract

Telomeres, the protective caps on the ends of chromosomes, offer a promising avenue for assessing life‐history trade‐offs, as they shorten in response to stressors and are predictive of the remaining lifespan. In addition to carrying out an experimental case study on a free‐living songbird, we conducted a systematic review and two meta‐analyses (28 studies, 54 effect sizes) exploring the associations between telomeres and (1) testosterone and (2) sexual ornamentation. In line with the findings of our case study, neither meta‐analysis showed an overall correlation of testosterone or sexual ornamentation with telomere length or telomere dynamics.

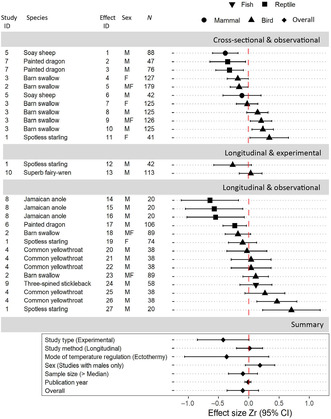

## 1 INTRODUCTION

Because resources are finite, life‐history theory predicts that organisms should prioritise resources for traits that increase fitness, thereby generating trade‐offs in the allocation of resources between current and future reproduction (Stearns, 1992). Sexual ornaments, such as vivid patches of colour, elongated tail feathers, and antlers or horns, are a form of reproductive investment that can play an important role in determining current reproductive success (Andersson, 1994). Sexual ornaments are complex traits that can convey information to prospective mates concerning fertility (Pomiankowski & Wedell, 2021; Rogers et al., 2008), paternal caregiving potential (Hill, 1992), or mate defence (Alatalo et al., 1991; Holmberg et al., 1989; Titman & Lowther, 1975), and the same ornament can also serve as a signal to competitors (Grether, 1996; Ligon et al., 1990; von Schantz et al., 1989). Ornament honesty is thought to be maintained by the fitness costs of ornamentation (Grafen, 1990; Zahavi, 1977). Such costs can arise in a variety of ways: the striking colouration that attracts a mate may also attract the unwanted attention of predators (Swierk et al., 2021; Wuthrich et al., 2022); the elaborate display that signals quality and condition to the opposite sex may also invite intrasexual competition (Madden, 2002; Pestana et al., 2018); and a stand‐out adornment may be carried at the expense of immunocompetence (Verhulst et al., 1999). These consequences, in turn, can result in reduced somatic condition and shortened lifespan, thereby decreasing future reproduction (Grether, 1997; Simons et al., 2016).

Telomeres are a promising method of assessing the cumulative physiological costs that mediate life‐history trade‐offs and may act as an integrative biomarker that broadly reflects an individual's investment in self‐maintenance (Monaghan & Haussmann, 2006; Young, 2018). Telomeres are the highly conserved, noncoding, protective nucleoprotein caps on the ends of eukaryotic chromosomes that aid in preventing damage to the coding regions of DNA (Blackburn, 2005). Telomeres shorten in response to physiological stress (Chatelain et al., 2019; Reichert & Stier, 2017) via higher oxidative stress (Armstrong & Boonekamp, 2023) and increased cell division through growth or cell renewal. Thus, as telomere lengthening mechanisms are downregulated in somatic tissues, telomere length tends to decline with age (Remot et al., 2021). Rates of telomere shortening are highest early in life, in concordance with the greatest period of growth and development (Hall et al., 2004; Sheldon et al., 2022). Telomere loss during adulthood, while significant, is often much smaller in magnitude (Salomons et al., 2009; Spurgin et al., 2018; Vedder et al., 2017). As a consequence, early‐life conditions exhibit an outsized effect on telomere length at sexual maturity (Boonekamp et al., 2014; Eastwood et al., 2023,b; van Lieshout et al., 2021). Thus, in comparison with telomere length (measured at a single time point), of greater relevance in the study of telomeres in adulthood are telomere dynamics (measured over multiple time points). As would be expected for a trait that broadly reflects a somatic state, telomere dynamics in adult birds have been shown to predict remaining lifespan (Barrett et al., 2013; Salomons et al., 2009; Sheldon et al., 2022). A recent review found that a majority of experimental studies have shown that reproduction increases the rate of telomere shortening (Sudyka, 2019). We might therefore expect that producing and displaying a sexual ornament will result in telomere shortening.

In testing for the responses of telomeres to investment in sexual ornamentation, it is important to consider the physiological mechanisms that underpin ornament production, as these may not only mediate life‐history trade‐offs (Peters, 2007) but also may directly affect telomere length. In vertebrate males, the production of sexual ornaments is often controlled by testosterone (Hau, 2007), the primary male sex hormone. In addition to its role in sexual ornament production, testosterone has been shown to regulate the activity of telomerase, the enzyme responsible for maintaining telomere length (Chan & Blackburn, 2004; Greider & Blackburn, 1985). Testosterone's relationship with telomerase exhibits high variability, being capable of both upregulating (Bär et al., 2015; Chen et al., 2012) and downregulating (Meeker et al., 1996; Ravindranath et al., 2001) telomerase activation. Testosterone may also play an indirect role in telomere dynamics by regulating behaviour (Lynn, 2008), immunity (Folstad & Karter, 1992; Foo et al., 2017), glucocorticoid secretion (Roberts et al., 2004), and metabolism (Buchanan et al., 2001; Navara et al., 2006), all of which have been associated with reduced telomere length (Angelier et al., 2018; Armstrong & Boonekamp, 2023; Chatelain et al., 2019).

The majority of studies that have examined the relationship between testosterone or sexual ornamentation and telomeres have been observational, which is problematic for quantifying the importance of trade‐offs as individual heterogeneity can obscure trade‐offs at the population level (Bolund, 2020; Stearns, 1989; van Noordwijk & De Jong, 1986). Only two longitudinal studies experimentally manipulated testosterone levels or sexual ornament expression in free‐living, sexually mature individuals, and with mixed results. While testosterone treatment increased telomere shortening rate in dark‐eyed juncos (*Junco hyemalis*; Heidinger et al., 2021), reduction of ornamental throat feathers did not significantly affect telomere shortening in spotless starlings (*Sturnus unicolor*; Azcárate‐García et al., 2020), highlighting the need for further research. Accordingly, to assess telomere dynamics as a biomarker of the somatic costs of testosterone and sexual ornamentation, we used testosterone implants to experimentally induce the production of a sexual ornament in an Australian songbird, the superb fairy‐wren (*Malurus cyaneus*), and subsequently measured the effect on changes in telomere length. In this socially monogamous songbird, males compete for extra‐pair paternity (>60% of young; Brouwer et al., 2014) through the timing of the seasonal moult into a conspicuous breeding plumage. Several previous studies demonstrated that testosterone, and the early moult into and presence of the breeding plumage, are costly (McQueen et al., 2017; McQueen, Delhey, Szecsenyi, et al., 2021; Peters, 2000), and we therefore hypothesised that testosterone‐implanted, early‐moulting individuals would exhibit a sharper decline in telomere length than controls. Additionally, to summarise our current understanding of the topic and to identify knowledge gaps, we used meta‐analyses to assess the associations between telomere dynamics and (1) testosterone and (2) sexual ornamentation across vertebrates. We further conducted meta‐regressions to identify the elements of study design that may influence the magnitude and direction of these relationships.

## 2 MATERIALS AND METHODS

### 2.1 Study species and population

The superb fairy‐wren is a small (10 g) songbird native to eastern and southeastern Australia. This species is socially monogamous but exhibits high (>60% of offspring) rates of extra‐pair paternity (Brouwer et al., 2014). Superb fairy‐wrens are seasonally sexually dimorphic, with females brown year‐round while males moult into a conspicuous bright‐blue and black breeding plumage. The timing of this pre‐breeding moult is extremely variable, occurring late in spring in first‐year males and progressively earlier with age (Dunn & Cockburn, 1999; van de Pol et al., 2012), with no evidence for senescence (Cooper et al., 2021). Colour (reflectance) of male plumage is unrelated to male quality or timing of moult (McQueen, Delhey, Barzan, et al., 2021). Instead, moult timing strongly predicts male extrapair paternity: few males (~5%) will moult into blue plumage very early (before mid‐winter), and these will sire nearly half of all extra‐pair young (Dunn & Cockburn, 1999). Testosterone causes moult initiation and maintenance of early blue plumage, as shown observationally and experimentally (Peters et al., 2000). Early moult is associated with known costs: experimental testosterone treatment is immunosuppressive (in captive males; Peters, 2000) and early moult experimentally induced by testosterone treatment results in increased ectoparasite load and reduced fat reserves (in free‐living males; McQueen, Delhey, Szecsenyi, et al., 2021). Moreover, males in breeding plumage are likely at higher predation risk: they spend more time being vigilant against predators, and less time foraging (McQueen et al., 2017).

Data collection was carried out at Lysterfield Park (37.95°S, 145.30°E), an open eucalyptus woodland 30 km east of Melbourne, Australia, on the traditional land of the Bunurong and Wurundjeri People of the Eastern Kulin Nation. Field work was conducted from May to November in 2016 and 2017. It is during this time of year—from late autumn, through winter, and into early spring—that males moult into breeding plumage. All males included in our study are a subset of the individuals included in McQueen, Delhey, Szecsenyi, et al. (2021). They were captured via mistnetting, and each fitted with a unique combination of leg bands, including one numbered metal band (ABBBS authority nos. 2230, 3288), for identification of individuals in the field. Age class was determined from bill and plumage colouration (Higgins, 2001) and banding history in order to distinguish first‐year males (hereafter, “juveniles”) from males that are older than 1 year (“adults”). The age range within the “adult” age class was likely quite broad: mean male lifespan in superb fairy‐wrens is 3.44 years (Cooper, 2020), with a maximum of 12 years (Cooper et al., 2021). All field work was undertaken with approval from the Department of Biological Sciences Ethics Committee (BSCI/2013/10, BSCI/2016/03) and the Department of Land, Water & Planning (DEWLP permit no. 10007370).

### 2.2 Experimental protocol

Control implants (20 ± 0.2 mg) were composed of beeswax (Enkaustikos USP) and hardened peanut oil (Sigma). Treatment implants additionally contained 1 mg of powdered testosterone (T1500, Sigma). This dosage level was selected based on observed (unmanipulated) testosterone levels of the superb fairy‐wren (Peters et al., 2000, 2001), hormone implant dosages used in other studies of small birds (Quispe et al., 2015; W. Goymann and C. Villavicencio, personal communication), and superb fairy‐wren body size. Age class was determined at implantation; within each age class, study males were alternatingly assigned to either the treatment (T) group or the control (C) group. Males that were implanted in 2016 were not re‐implanted in 2017. T‐males (*n* = 24 males; 12 adults and 12 juveniles) received a subdermal treatment implant, from which testosterone was gradually released (Boersma et al., 2022), which results in a transient (15–32 days) increase in T levels just above the maximum recorded for this species and subsequently induces early breeding plumage moult (Peters et al., 2000). C‐males (*n* = 16 males; 4 adults and 12 juveniles) received an empty implant. All males were blood sampled on initial capture, and blood samples were stored in 70% ethanol at 4°C. All males were recaptured. Upon recapture, additional blood samples were collected: 16 males were sampled twice, 15 males were sampled three times, and 9 males were sampled four times. Mean ± SD interval between between first and last capture was 68 ± 46 days. T‐ and C‐males did not differ in implantation date (*t*‐test, *p* = .60) or recapture date (*t*‐test, *p* = .10). The interaction between implant type and age class was not a significant predictor of survival to the following year (McQueen, Delhey, Szecsenyi, et al., 2021).

### 2.3 Moult observation

Following implantation, all males were regularly resighted to determine moult timing. Moult progress was scored as % visible breeding plumage, estimated in increments of 5% (5% = a few blue and black feathers, 95% = a few brown feathers remaining in the black and blue breeding plumage; McQueen, Delhey, Szecsenyi, et al., 2021). Because not all males could be observed on the day that moult was completed, moult completion date was scored in 10‐day intervals. For this study, moult completion date is more relevant than the date of moult initiation, because males do not begin displaying to females until they have completed their moult (Green et al., 2000; Mulder, 1997).

### 2.4 Telomere length measurement

To measure telomere length, we used a quantitative polymerase chain reaction (qPCR) method based on Criscuolo et al. (2009) and validated in a closely related species (*Malurus coronatus*; Eastwood et al., 2018). DNA was extracted using a QIAamp DNA kit (Qiagen) and was automated using a QIAcube HT instrument. To ensure only high‐quality DNA was used for the qPCR assay we assessed DNA concentration and purity using a NanoDrop (ND‐1000) and ran each sample on a 1.5% agarose gel for confirming high molecular weight DNA. DNA from each sample was extracted once and run in duplicate on 96‐well plates, as well as being run on 1–4 additional plates. Implant types (T or C) were approximately equally represented on each plate. Subsequent samples of individuals were often, though not always, run on different plates. The telomere assay and control gene, glyceraldehyde‐3‐phosphate dehydrogenase (GAPDH), were run on separate plates. Total reaction volume was 15 μL and included 18–20 ng DNA, 7.5 μL SYBR Master Mix (Roche), and 300 nm of each GAPDH primer (GT2‐GAPDH‐forward 5′ – CCA TCA CAG CCA CAC AGA AG – 3′ and GT2‐GAPDH‐reverse 5′ – TTT TCC CAC AGC CTT AGC AG – 3′ (Atema et al., 2013)) or 500 nm of both telomere primers (Tel1b 5′ – CGG TTT GTT TGG GTT TGG GTT TGG GTT TGG GTT TGG GTT – 3′ and Tel2b 5′ – GGC TTG CCT TAC CCT TAC CCT TAC CCT TAC CCT TAC CCT – 3′). On each plate in duplicate, we included a no template control (nuclease free water, Ambion) and an inter‐plate control made up of multiple superb fairy‐wren individuals of different age classes. Telomere reactions were run using a LightCycler 480 qPCR instrument (Roche) at 95°C for 15 min, followed by 25 cycles of 15 s at 95°C, 30 s at 56°C, and 30 s at 72°C. GAPDH reactions were run at 95°C for 15 min, followed by 40 cycles of 15 s at 95°C, 30 s at 60°C, and 30 s at 72°C. A visual assessment of the melt curves confirmed that the intended targets were amplified.

The program LinRegPCR (version 2016) was used to calculate individual well qPCR efficiencies and Cq values (Ruijter et al., 2009). Telomere or GAPDH within‐plate duplicates with Cq values greater than 0.5 apart were excluded from analysis. After quality control, well efficiencies were 1.94 ± 0.05 (mean ± SD) for telomeres and 1.94 ± 0.03 for GAPDH. Relative telomere length (rTL) was calculated following Pfaffl (2001). Duplicate values of telomere length and GAPDH were averaged before dividing telomere length by GAPDH to produce our final rTL values. Our final data set consisted of 113 telomere estimates from 40 males. Inter‐assay repeatability of rTL was moderate (0.73), as calculated using the rptR() function (Stoffel et al., 2017), with rTL as the response variable and a concatenation of DNA sample ID as the random effect. Within‐plate repeatability was 0.87 for telomere Cq and 0.88 for GAPDH Cq, as calculated using Cq as the response variable and a concatenated “DNA sample ID by Run” as the random effect.

### 2.5 Statistical analysis

Data analysis was conducted in R (version 4.2.0; R Core Team, 2021). Student's *t*‐tests were conducted to compare pre‐implantation rTL values between T‐males and C‐males, as well as between adults and juveniles within each treatment group. We also used Student's *t*‐tests to compare post‐implantation testosterone levels between T‐males and C‐males, using testosterone data from McQueen, Delhey, Szecsenyi, et al. (2021), for the subset of males included in our study. All continuous variables were mean‐centred to allow for easier interpretation of main effects included in interactions. Models were run using the lmer function from the lme4 package (Bates et al., 2015) with restricted maximum likelihood estimation. To account for the effects of qPCR run on rTL estimates and to combine repeated measures into a single value for analysis (Eastwood et al., 2018), we ran a linear mixed‐effects model with rTL as the response variable, “DNA sample ID” as a fixed effect, and “Plate ID” as a random effect. The model estimates (predicted values) produced by this model were *Z*‐scored to facilitate interpretation of effect sizes, comparison across studies, and inclusion in meta‐analyses (Verhulst, 2020). These *Z*‐scored rTL values were used as the response variable in subsequent analyses. We constructed a linear mixed effects model to test the prediction that testosterone implants would accelerate telomere shortening, controlling for variation in the time since implantation. Age class, implant type (control or testosterone), the number of days since implantation (sampling interval), and the interaction between implant type and sampling interval were included as fixed effects, and male ID as a random effect to account for the non‐independence of repeated sampling of individuals. Age class (juvenile or adult) and its interaction with sampling interval were also included as fixed effects to control for possible age differences in speed of telomere length attrition (Roast et al., 2022; Sheldon et al., 2022). The interaction between age class and sampling interval was non‐significant (*p* = .124), and was removed from the final model. rTL was not significantly different between 2016 and 2017 (*t*‐test, *p* = .94), and year was therefore not included in the model. Intra‐individual repeatability was calculated using the rptR() function (Stoffel et al., 2017). In all analyses, we confirmed that the model assumptions were satisfied via visual inspection of residual plots.

### 2.6 Meta‐analyses and meta‐regressions

**FIGURE A1:**
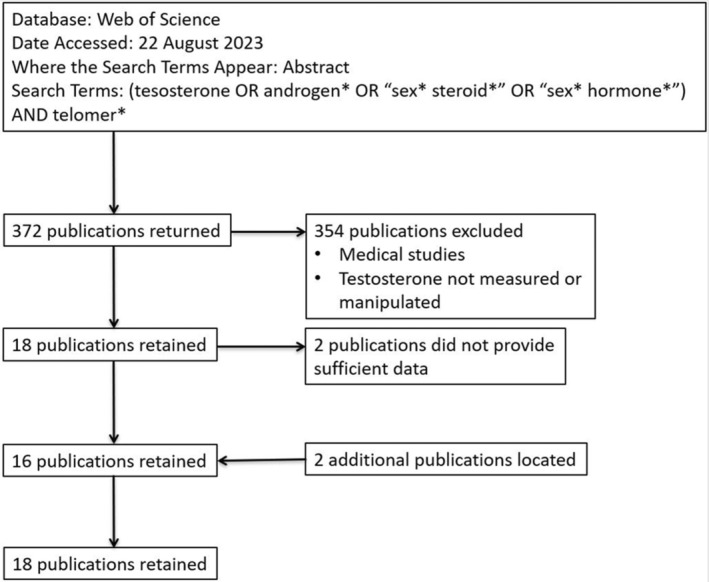
A PRISMA flow chart of the selection process for our literature search investigating the interaction between testosterone and telomeres. Sample sizes do not include our case study on superb fairy‐wrens.

**FIGURE A2:**
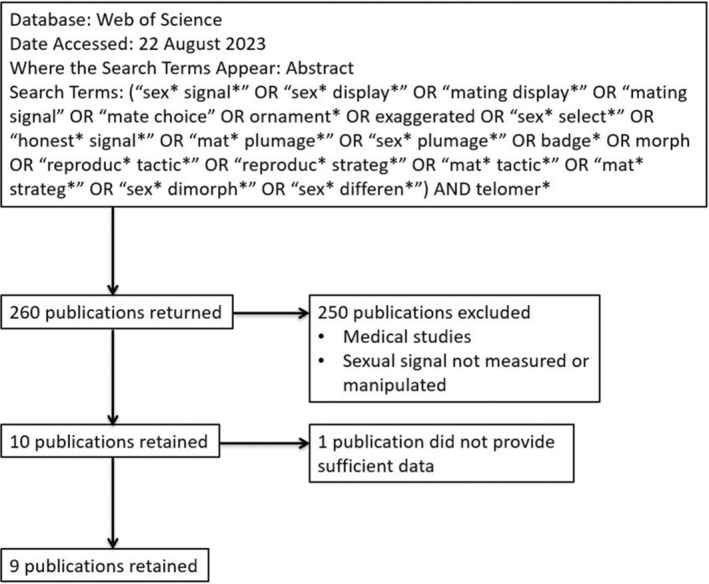
A PRISMA flow chart of the selection process for our literature search investigating the interaction between sexual ornamentation and telomeres. Sample sizes do not include our case study on superb fairy‐wrens.

We conducted two systematic searches of the literature (following the PRISMA recommendations; Moher et al., 2009) using Web of Science on 22 August 2023. We used the search string (testosterone OR androgen* OR “sex* steroid*” OR “sex* hormone*”) AND telomer* to identify studies that had assessed the relationship between telomere length or dynamics and testosterone (Figure A1). We then used the search string (“sex* signal*” OR “sex* display*” OR “mating display*” OR “mating signal” OR “mate choice” OR ornament* OR exaggerated OR “sex* select*” OR “honest* signal*” OR “mat* plumage*” OR “sex* plumage*” OR badge* OR morph OR “reproduc* tactic*” OR “reproduc* strateg*” OR “mat* tactic*” OR “mat* strateg*” OR “sex* dimorph*” OR “sex* differen*”) AND telomer* to identify studies that had assessed the relationship between telomere length or dynamics and sexual ornaments (Figure A2). The former returned 372 publications, and the latter returned 260 publications. We removed duplicates, biomedical studies (including those on non‐human species), non‐empirical studies (e.g., reviews, meta‐analyses, or theoretical publications), studies that did not measure telomere length or dynamics and either testosterone levels or sexual ornament expression, and studies that did not provide sufficient information to determine effect sizes. After additional searches located two more testosterone studies, we retained 18 studies that assessed telomere length or dynamics in relation to testosterone (see Figure 2 for a list of the cited studies) and 9 studies that assessed telomere length or dynamics in relation to sexual ornaments (Figure 3).

Data were either extracted from text or tables, or from figures using webplotdigitizer (Rohatgi, 2021). For studies in which a lack of information prevented us from standardising effect sizes, we contacted the publication authors (three in total), and received the necessary data from one of these. Overall, we were able to extract a total of 54 effect sizes from 28 studies, including our case study on superb fairy‐wrens. For study species in which the males occur as distinct morphs, with each morph expressing a discrete level of ornamentation, we used the two most disparate morphs (representing the lowest and highest levels of investment in the ornament) to determine the association between telomere length or dynamics and sexual ornamentation. Because telomere length commonly decreases with age, while ornamentation often also varies with age, effect sizes from cross‐sectional studies that did not control for age (*n* = 1 effect size) were excluded from our analyses. Effect size sign (+/−) was reversed (i) when increasing ornament investment (e.g., ornament colour) was denoted by decreasing numerical values, and (ii) when the response variable, instead of being TL or ΔTL, was “rate of TL loss.” For cases in which it was not possible to determine the direction (+/−) of an effect size, that effect size was excluded (*n* = 1). When effect sizes were provided separately by sex, we present them here separately. For cases in which the effect size for one sex was irrelevant to our purposes (e.g., when females lack the sexual ornament), that effect size was excluded. For details, see Figures 2 and 3. Effect sizes were converted to Pearson's correlation coefficient (*r*), and then, using Fisher's transformation, to *Zr*, following equation 20 in Nakagawa and Cuthill (2007). The standard error of *Zr* was calculated following Nakagawa and Cuthill (2007) and converted to variance.

Meta‐analyses were conducted using the metafor package (version 3.4.0; Viechtbauer, 2010) in R. For each analysis, we used a multilevel random‐effects model that included *Zr* and the variance of *Zr* as the response variables. Study ID was included as a random effect in all models. Effect size ID was included as a random effect to assess the within‐study variance. To account for multiple studies using the same study species, we additionally included Species ID as a random effect. We ran this model (null model) without moderators to determine the meta‐analytic effect size and between‐study heterogeneity (*Q* and $I_{\text{total}}^{2}$). To account for the potential influence of phylogenetic relatedness, we created a cladogram using the rotl package (Michonneau et al., 2016), and determined branch lengths using the compute.brlen() function from the ape package (Paradis & Schliep, 2019). Phylogeny was then run in a separate model as a random effect. In both the null model and the phylogenetic model, we assessed the proportion of heterogeneity that was explained by each random effect, and used the change in AIC_c_ to assess whether including phylogeny improved model fit; if ΔAIC_c_ was <−2.00, the phylogenetic model was considered to have improved model fit.

In our meta‐regressions we included as moderators: sex (a two‐level factor: studies that only included males vs. those that included both sexes or only females; sample sizes did not permit splitting this up further); study type (observational vs. experimental); and study method (cross‐sectional vs. longitudinal). Study type (observational vs. experimental) was included as experimental methods are better able to detect life‐history trade‐offs (Bolund, 2020; Stearns, 1989; van Noordwijk & De Jong, 1986). Study method (cross‐sectional vs. longitudinal) was included in order to separately assess the associations of telomere length (measured in cross‐sectional studies) and the associations of telomere dynamics (measured in longitudinal studies) with testosterone and sexual ornamentation. In order to assess the potential role of publication year and study size in introducing sampling bias, we included mean‐centred year, as well as sample size (as a two‐level factor: ≤ median or > median) as moderators. In our meta‐regressions for the studies on sexual ornaments, we additionally included mode of temperature regulation (endotherm or ectotherm) as a moderator. We did not include this in our meta‐regressions of the testosterone studies because those studies were conducted solely on endotherms. We did not include telomere measurement method (qPCR vs. TRF) as a moderator in either meta‐analysis because of the low number of studies that used TRF (testosterone vs. telomeres: *n* = 2; sexual ornamentation vs. telomeres: *n* = 1), and because, within each meta‐analysis, TRF was only used for a single species. The variance inflation factor was calculated to check for collinearity among moderators.

## 3 RESULTS

### 3.1 Case study

#### 3.1.1 Testosterone treatment had no effect on telomere length or dynamics in superb fairy‐wrens

Testosterone treatment advanced moult completion, with T‐males completing their moult 51 ± 9 days earlier (mean ± SE; *t*‐test, *p* < .001) than C‐males. These values are very similar to the results of Peters et al. (2000), on which our study design was based. Among T‐males, adults moulted 33 ± 8 days earlier than juveniles (*p* < .001), and were generally the earliest‐moulting males in our study population. Among C‐males, adults moulted 35 ± 16 days earlier than juveniles (*p* = .03).

From 15 to 32 days post‐implant, testosterone levels in C‐males remained low (0.177 ± 0.018 ng/mL). Testosterone levels in T‐males were elevated (5.9 ± 1.6 ng/mL). This is above the maximum testosterone level recorded for this species (3.7 ng/mL; Peters et al., 2000) but within the range observed in other *Malurus* fairy‐wrens (Enbody et al., 2018; Lindsay et al., 2011). Owing to the limited collection of pre‐implantation testosterone samples (*n* = 5), we were unable to test whether pre‐implantation testosterone levels differed between T‐males and C‐males. However, we did find that testosterone levels in T‐males did not differ from those of C‐males following moult completion (*t*‐test, *p* = .75).

**FIGURE 1:**
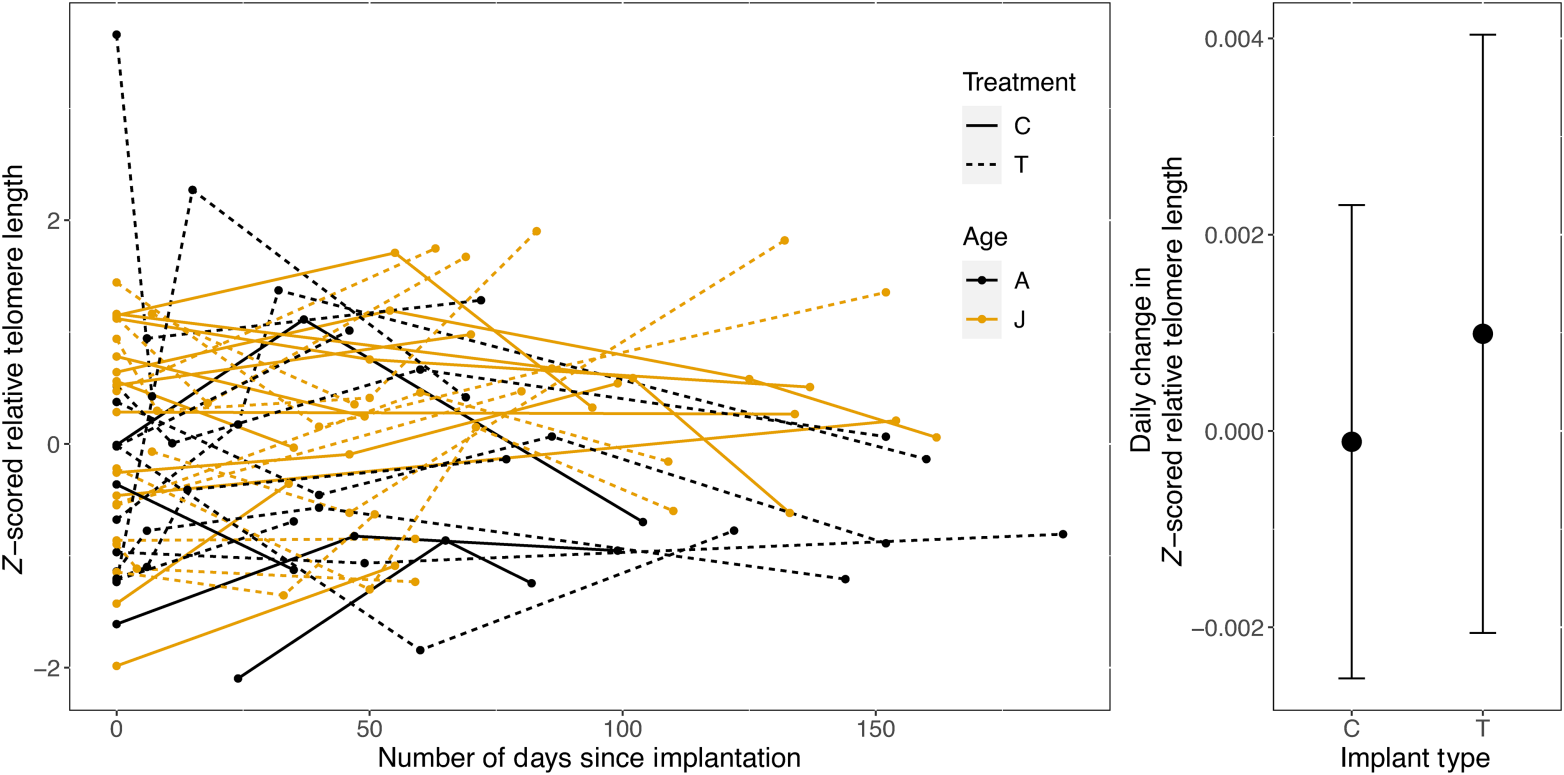
Telomere dynamics following implantation (Day 0) did not vary between control (C)‐ and testosterone (T)‐implanted males or by age class (adults, “A”, aged 2 years and above; and juveniles, “J”, in their first moult season). The “adult” age class exhibited shorter telomeres than the “juvenile” class (estimate ± SE: −0.425 ± 0.278 rTL SD), and overall, telomere length declined with number of days since implantation (−0.00011 ± 0.0024 rTL SD per day), but these differences were not significant. Although the effect of treatment over time was also not significant, the effect size was positive (0.0011 ± 0.0030 rTL SD per day), which is in the opposite direction to what we hypothesised.

Pre‐implantation rTL was not significantly different between T‐males and C‐males (mean ± SE; T‐males: −0.041 ± 1.076; C‐males: 0.057 ± 0.917; *t*‐test, *p* = .78). rTL did not vary significantly with age, implant type, sampling interval, or the interaction between implant type and sampling interval (all *p* > .05; Table 1; Table A1; Figure 1). The effect of days since implantation was negative for C‐males (−0.017 rTL SD across the sampling range, 150 days) but in testosterone treated males, this was positive (0.155 rTL SD for T‐males across the sampling range), which is opposite to the hypothesised direction. rTL tended to be shorter by 0.425 SD in adults (aged 2 years and above) compared to juveniles (1 year old), although this difference was not significant (*p* = .135; Table 1). The considerable magnitude of this difference may be due to the high likelihood that the age class “adults” contained a wide range of ages: mean male lifespan in this species is 3.44 years (Cooper, 2020); maximum lifespan is 12 years (Cooper et al., 2021). Within‐individual rTL repeatability was 0.51 (Table 1), close to the average for qPCR‐based telomere studies (Kärkkäinen et al., 2022).

**TABLE 1:** Telomere dynamics were not affected by testosterone treatment in male superb fairy‐wrens.

Fixed effects	Estimate (SE)	*t*	*p*
Intercept	−0.130 (0.217)	−0.600	.553
Age class (adult)	−0.425 (0.278)	−1.529	.135
Sampling interval	−0.00011 (0.0024)	−0.048	.962
Implant type (T)	0.220 (0.280)	0.787	.436
Implant type (T) × Sampling interval	0.0011 (0.0030)	0.377	.707
**Random effects**	**Variance**		
Male ID	0.505		
Residual	0.523		
	**Marginal *R*** ^**2**^	**Conditional *R*** ^**2**^	
	.043	.513	

*Note*: Shown is the linear mixed‐effects model testing for the effect of testosterone treatment on *Z*‐scored rTL estimates over time. Age class is “adult” (aged 2 years and above) or “juvenile” (reference category; aged 1 year). Sampling interval was mean‐centred and refers to the number of days since implantation; i.e., effect size is per day. Implant type is either “T” or “C” (reference category). See Table A1 for the full model, with all non‐significant interactions included.

### 3.2 Meta‐analysis 1: Testosterone versus telomeres

**FIGURE 2:**
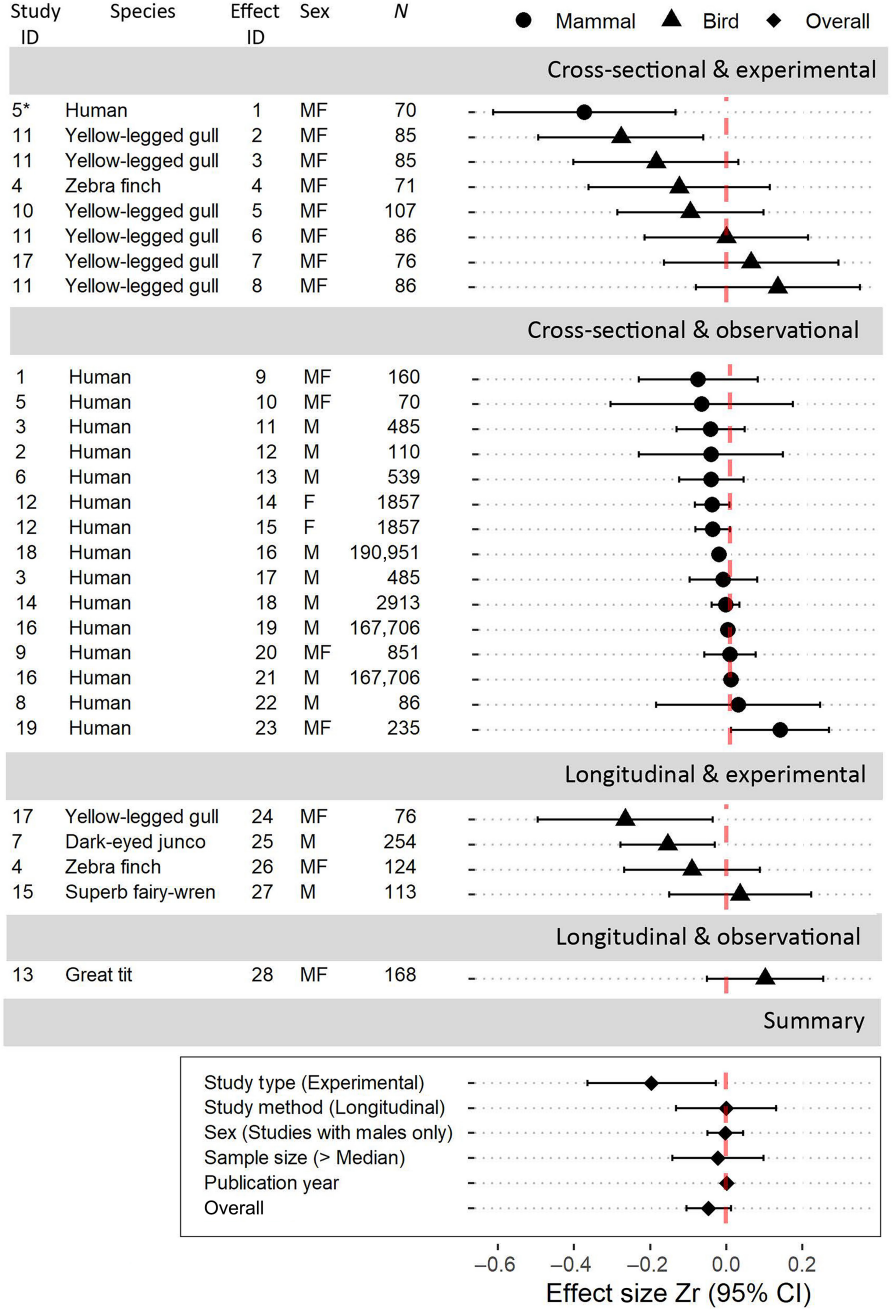
The overall meta‐analytic mean of the association between testosterone and telomeres was negative (OVERALL refers to the meta‐analytic mean effect size produced by the null model), though the confidence interval slightly overlapped zero. Moderator effects are shown under “Summary.” Only study type (observational vs. experimental) was a significant moderator, with experimental studies producing more strongly negative effect sizes. For each moderator, Zr shows the standardised effect of that moderator on a given effect size. Reference categories are as follows: Study Type (Observational), Study Method (Cross‐Sectional), Sex (Studies Also Including Females), and Sample Size (≤ Median (118.5)). For each effect size, Zr shows the standardised correlation between testosterone and telomere length or dynamics. *N* refers to the number of telomere samples; M = male only, F = female only, MF = males and females included in the effect size. Study ID with associated citation: (1) Al‐Thuwaini (2022); (2) Bekaert et al. (2005); (3) Coburn et al. (2018); (4) Criscuolo et al. (2020); (5) Drury et al. (2014); (6) Gu et al. (2020); (7) Heidinger et al. (2021); (8) Huang et al. (2019); (9) Needham et al. (2014); (10) Parolini et al. (2018); (11) Parolini et al. (2019); (12) Song et al. (2018); (13) Stier et al. (2015); (14) Yeap et al. (2019); (15) Taylor et al. (this study); (16) Marriott et al. (2023); (17) Noguera et al. (2022); (18) Codd et al. (2021); (19) Axson et al. (2018). *In study 5, one effect size was of stress‐induced testosterone levels, as opposed to baseline testosterone.

Of 28 effect sizes derived from 19 studies, including our case study on superb fairy‐wrens, 21 were negative and 7 were positive (Figure 2). The overall effect size of testosterone on telomere length or dynamics was negative, though confidence intervals slightly overlapped zero (meta‐analytic mean [95% CI]: −0.045 [−0.103, 0.013]). Null model heterogeneity was high ($I_{\text{total}}^{2}$ = 97.21%; *Q*_(df = 27)_ = 138.3559, *p* < .0001; Table A2), indicating a high amount of variance among studies, which was largely explained by Species ID (*I* ^2^ = 87%). Phylogenetic variance did not explain any of the heterogeneity when included in the null model and did not improve model fit (ΔAIC_c_ = +2.00; Table A3). Study type (observational or experimental) was a significant moderator, with experimental studies producing more negative effect sizes (mean [95% CI]: −0.195 [−0.364, −0.027]; *p* = .023; Table A4). Sex, sample size, and publication year were not significant moderators (Table A4). Study method (cross‐sectional or longitudinal) was also not a significant moderator, indicating that the strength and direction of the association between testosterone and telomere length is not significantly different from that of the association between testosterone and telomere dynamics.

### 3.3 Meta‐analysis 2: Sexual ornaments versus telomeres

**FIGURE 3:**
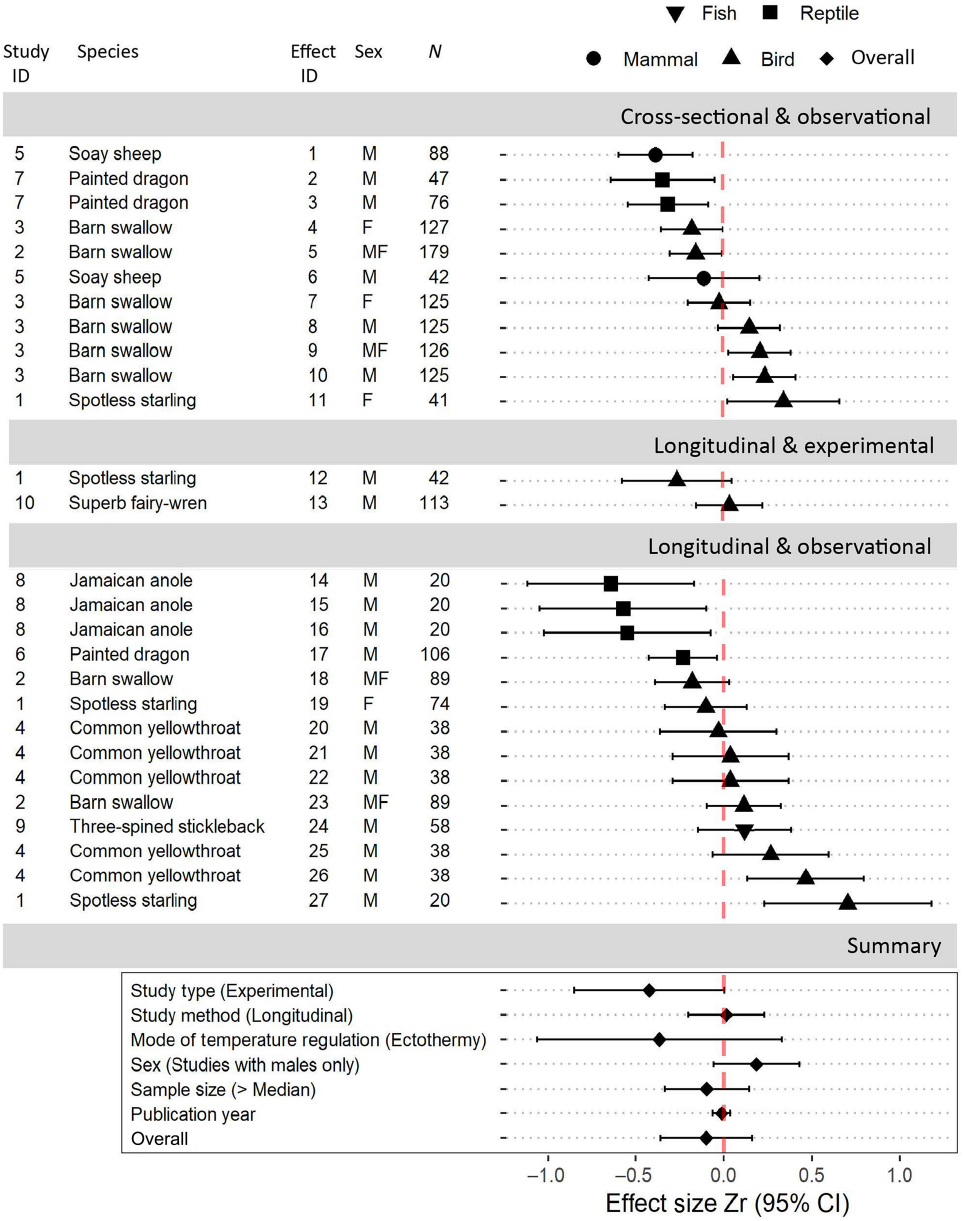
The overall meta‐analytic mean of the association between sexual ornamentation and telomeres was slightly negative (see OVERALL, which shows the mean meta‐analytic effect size), though confidence intervals overlapped zero. Moderator effects are shown under “Summary.” Only study type (observational vs. experimental) was a near‐significant moderator, with experimental studies producing more strongly negative effect sizes. For each moderator, Zr shows the standardised effect of that moderator on a given effect size. Reference categories are: Study Method (Cross‐Sectional), Mode of Temperature Regulation (Endothermy), Sex (Studies Also Including Females), and Sample Size (≤ Median (Kempenaers et al., 2008)). *N* refers to the number of telomere samples collected. M = male only, F = female only, MF = males and females included in the effect size. For studies in which the results were reported separately for males and females (Study ID 3), we present these separately here. For studies in which data for the female were not applicable (because the females of that species do not exhibit sexual ornaments; Study ID 9), these were excluded. When the direction (+/−) of an effect size was not possible to determine, that effect size was excluded (Study ID 9). When increasing ornament investment (e.g., ornament colour) was denoted by decreasing trait values (Study 3, Effect IDs 7 and 10), the sign of the effect size (+/−) was reversed. For studies in which the response variable, instead of being TL or delta TL, was “rate of TL loss” (Study ID 1, Effect ID 12 and 27) the sign of this effect size (+/−) was reversed. Study ID with associated citation: (1) Azcárate‐García et al. (2020); (2) Kauzálová et al. (2022); (3) Parolini et al. (2017); (4) Taff et al. (2017); (5) Watson et al. (2017); (6) Giraudeau et al. (2016); (7) Rollings et al. (2017); (8) Figueiredo Passos et al. (2022); (9) Álvarez‐Quintero et al. (2021); (10) Taylor et al. (this study).

Of 27 total effect sizes derived from 10 studies, 15 were negative and 12 were positive (Figure 3). One study, in addition to our case study on superb fairy‐wrens, manipulated the sexual ornament: in a free‐living population of spotless starlings, the ornament was altered by clipping a patch of elongated feathers (Azcárate‐García et al., 2020). That study examined changes in telomere length over time, but did not find an effect of the manipulation on telomere dynamics.

The overall effect size of sexual ornament expression on telomere length or dynamics was slightly negative, but overlapped with zero (meta‐analytic mean [95% CI]: −0.085 [−0.257, 0.087]; Table A5). Null model heterogeneity was high ($I_{\text{total}}^{2}$ = 81.92%; *Q*_(df = 26)_ = 100.2855, *p* < .0001). Phylogenetic variance explained a majority of this heterogeneity (*I* ^2^ = 67%), but its inclusion did not improve model fit (ΔAIC_c_ = −1.38; Table A6). Study type (observational or experimental) was the only near‐significant moderator (*p* = .051), with experimental studies producing more negative effect sizes. Mode of temperature regulation (endotherm or ectotherm), sex, sample size, and publication year were not significant moderators (Table A7). Study method (cross‐sectional or longitudinal) was also not a significant moderator, indicating that the strength and direction of the association between sexual ornamentation and telomere length is not significantly different from that of the association between sexual ornamentation and telomere dynamics.

## 4 DISCUSSION

We tested whether costs assumed to be associated with producing, maintaining, and/or displaying a sexual ornament are reflected in shorter telomere length or faster telomere attrition. To this end, we first conducted a case study on free‐living male superb fairy‐wrens. We used experimental testosterone treatment to stimulate the production of a sexual ornament, early moult to blue breeding plumage, but we found no increase in the rate of telomere shortening. We additionally conducted meta‐analyses on the associations between telomere length or dynamics and testosterone or sexual ornamentation. These analyses produced similar results, showing no evidence for a significant association with telomere length or dynamics. We here discuss potential explanations for the apparent absence of a relationship between telomeres and testosterone or sexual ornamentation.

### 4.1 Case study in the superb fairy‐wren

We found no evidence for an acceleration of telomere shortening due to testosterone treatment, with a positive but non‐significant effect of the treatment (Table 1, Figure 1). This is not a consequence of ineffectiveness of the treatment or lack of physiological costs: testosterone implants raised testosterone levels which caused T‐treated males to moult into blue breeding plumage 8 weeks earlier than C‐males. Previous studies, which also used testosterone implants to stimulate early moult to breeding plumage, showed that forced early moult results in reduced immune system function, decreased body fat, and increased ectoparasite load (McQueen, Delhey, Szecsenyi, et al., 2021; Peters, 2000). Furthermore, males in breeding plumage exhibit reduced time spent foraging, and increased time spent being vigilant against predators (McQueen et al., 2017). Thus, while our results may reflect a true absence of an effect of elevated testosterone and/or increased sexual signalling on telomere dynamics, the lack of a statistically significant negative effect may also be attributable to insufficient statistical power. Limited statistical power may be due, for example, to limited sample size (*n* = 113 samples from *n* = 40 males), or to resampling after a relatively short interval (0.17 years), before any costs of the extended display of the ornament had accumulated. The only study showing that experimental testosterone treatment resulted in accelerated telomere attrition in sexually mature individuals resampled a large number of males (*n* = 254 samples from *n* = 114 males) after a mean interval of 1.56 years (dark‐eyed juncos; standardised effect size: −0.00042 rTL SD per day, or −0.154 per year; Heidinger et al., 2021; Effect ID 23 in Figure 2). It is possible therefore that a detectable negative effect of testosterone on telomere attrition would appear if we had resampled males after a longer period, or repeated treatment over multiple years (although not necessarily; see e.g., Atema et al., 2022), or conducted our experiment in years with harsher environmental conditions that strongly affect the costs of early moult (van de Pol et al., 2012).

We used testosterone to experimentally induce the sexual ornament, as the two are inextricably connected in male superb fairy‐wrens (McQueen, Delhey, Szecsenyi, et al., 2021; Peters et al., 2000). One previous study assessed telomere dynamics in relation to the experimental manipulation of a sexual ornament (Azcárate‐García et al., 2020; Effect ID 12 in Figure 3). As in our study, that study found no significant response of telomere dynamics to ornament manipulation, but power was also limited in that study. Clearly, further experimental studies on other species with well‐understood costs of testosterone and/or sexual ornaments will be required to establish whether and under what conditions these traits influence telomere dynamics.

### 4.2 Meta‐analyses

While both estimated meta‐analytic effect sizes were negative as predicted, neither were significantly different from zero, with the confidence interval for the overall effect of testosterone only slightly overlapping zero (Figure 2), and the confidence interval for the ornament effect broadly overlapping (Figure 3). These results indicate that there is currently limited evidence for an association between testosterone or sexual ornamentation and telomere length (cross‐sectional studies comparing individuals) or dynamics (longitudinal studies within individuals). In addition to the large variance between studies preventing definitive statements on the relationship between telomeres and testosterone or sexual ornamentation, study design limitations might also play an important role.

Experimental studies produced larger effect sizes: study type (observational vs. experimental) was a significant moderator in the meta‐analyses on the relationship between telomeres and testosterone; it was near‐significant in the ornament meta‐analysis, possibly because there were only 2 effect sizes from experimental ornament manipulations. A clearer negative effect is strongly predicted in experimental studies of life‐history trade‐offs because by manipulating the trait or traits of interest, the influence of individual heterogeneity is removed. Contrastingly, in observational studies, depending on whether differences in resource acquisition or allocation among individuals prevail, at the population level, negative, positive, or no covariation among traits are all compatible with trade‐offs between these traits within individuals (van Noordwijk & De Jong, 1986). Thus, our analyses highlight the importance of further experimental studies that investigate the effect on telomere dynamics of manipulating testosterone and/or sexual ornaments.

Incorporating testosterone responsiveness may potentially be an informative direction for future experimental research, as suggested by Drury et al. (2014; Study 5 in Figure 2), who measured the relationship between telomere length and *stress‐induced* testosterone levels. Telomere length is a static trait (Kärkkäinen et al., 2022), changing slowly with increasing age; in comparison, testosterone levels vary strongly within‐ and between individuals both seasonally (e.g., a peak during breeding) and on shorter timescales (Kempenaers et al., 2008). Individual testosterone levels are dynamically shaped by individual social environment and experiences (Goymann et al., 2019), as well as many aspects of the non‐social environment (Goymann, 2009), including disease (Boonekamp et al., 2008). This complicates tests for associations with other traits. Responses to social or other challenges, or via experimental manipulations (e.g., through GnRH injections; Goymann, 2009), may provide more robust characterisation of individual variation in testosterone levels over time. Responses to challenges or manipulations may therefore be better suited than baseline testosterone levels to investigate how testosterone relates to telomere dynamics.

Phylogeny did not explain any of the variance the relationship between telomeres and testosterone, which suggests that the mechanism mediating testosterone's relationship with telomeres may be conserved across taxa. This would not be unexpected, as the structure of testosterone does not vary across vertebrates, and its major functions are highly conserved (Hau, 2007). In contrast, phylogeny explained much of the variance in the relationship between telomeres and sexual ornaments. This suggests that the relationship between sexual ornaments and telomere length or dynamics is more similar between closely related species. This may be a result of ornament type varying taxonomically: ornaments are highly variable among species, but more closely related species tend to have more similar ornaments (e.g., McGraw, 2005; Prager & Andersson, 2009). For example, among the species included in our meta‐analysis, nearly all studies on birds (in which the ornament was invariably plumage‐based, either plumage colour or morphology) produced positive or non‐significant results, while all studies on reptiles (in which the ornament was the colour of the integument) produced significant negative results. Unfortunately, limited representation of other classes in our meta‐analysis (mammals and fish), as well as limited representation of ornament diversity within classes, prevents us from more thoroughly investigating the role of ornament type in shaping the association between telomeres and sexual ornamentation. In addition to more studies on mammals and fish, we also wish to encourage studies of bird integumentary colouration or morphological ornaments in reptiles.

## 5 CONCLUSION

We found no evidence of shorter telomeres or increased telomere shortening as a cost of increased testosterone or sexual ornament display. In our case study on superb fairy‐wrens, our inability to detect an effect may be related to our sampling design. Our meta‐analyses show no association between telomere length or dynamics and testosterone or sexual ornamentation. We note however that there were very few longitudinal, experimental studies available for the meta‐analyses, and an increase of such studies may well lead to different conclusions.

## AUTHOR CONTRIBUTIONS

**Gregory T. Taylor:** Formal analysis (lead); writing – original draft (lead); writing – review and editing (lead). **Alexandra McQueen:** Conceptualization (lead); investigation (lead). **Justin R. Eastwood:** Formal analysis (equal); investigation (equal). **Andréaz Dupoué:** Investigation (equal). **Bob B. M. Wong:** Writing – review and editing (equal). **Simon Verhulst:** Formal analysis (equal). **Anne Peters:** Conceptualization (equal); formal analysis (equal).

## CONFLICT OF INTEREST STATEMENT

The authors declare no conflicts of interest.

## Data Availability

All data and code presented in this paper are available from the figshare repository: 10.6084/m9.figshare.24804864.

## Appendix

**TABLE A1:** Telomere dynamics were not affected by testosterone (T) treatment in male superb fairy‐wrens.

Dependent variable: *Z*‐scored rTL estimates			
Fixed effects	Estimate (SE)	*t*‐value	*p*‐value
Intercept	−0.129 (0.214)	−0.650	.520
Age (adult)	−0.446 (0.274)	−1.627	.113
Sampling interval	−0.0015 (0.0026)	−0.596	.553
Implant type (T)	0.236 (0.276)	0.855	.398
Age (adult) × Sampling interval	−0.0052 (0.0034)	−1.556	.124
Implant type (T) × Sampling interval	0.0037 (0.0035)	1.069	.289
**Random effects**	**Variance (SD)**		
Male ID	0.485 (0.696)		
	**Marginal *R*** ^**2**^	**Conditional *R*** ^**2**^	
	.057	.511	

*Note*: Shown is the linear mixed‐effects model testing for the effect of testosterone treatment on *Z*‐scored rTL estimates over time, including all non‐significant interactions. Age is “adult” (aged 2 years and above) or “juvenile” (reference category; aged 1 year). Sampling interval was mean‐centred and refers to the number of days since implantation; i.e., effect size is per day. Implant type is either “T” or “C” (control; reference category).

**TABLE A2:** The null model for the meta‐analysis of testosterone, including Study ID, Effect ID, and Species ID as random effects only, shows a non‐significant negative relationship between testosterone and telomere length or dynamics.

Fixed effects	Estimate	SE	*Z*	*p*	Lower CI	Upper CI
Intercept	−0.045	0.030	−1.525	.127	−0.103	0.013
**Random effects**	**Estimate**	***I*** ^**2**^				
Between study variance (Study ID)	0.0002	8.892				
Within study variance (Effect ID)	0	1.147				
Species variance (Species ID)	0.0023	87.168				

*Note*: Overall *I* ^2^ was 97.21%. *Q*_(df = 27)_ = 138.3559, *p* < .0001; AIC_c_ = −50.41099.

**TABLE A3:** As in the null model, the phylogenetic model shows a non‐significant negative relationship between testosterone and telomere length or dynamics; including phylogeny did not improve model fit (ΔAIC_c_ > −2.00).

Fixed effects	Estimate	SE	*Z*	*p*	Lower CI	Upper CI
Intercept	−0.045	0.030	−1.525	.127	−0.103	0.013
**Random effects**	**Estimate**	***I*** ^**2**^				
Between study variance (Study ID)	0.0002	8.892				
Within study variance (Effect ID)	0	1.147				
Species variance (Species ID)	0.0023	87.168				
Phylogenetic variance	0	0				

*Note*: Overall *I* ^2^ was 97.21%. *Q*_(df = 27)_ = 138.3559, *p* < .0001; AIC_c_ = −48.41009.

**TABLE A4:** The full meta‐regression model shows no relationship between testosterone and telomere length or dynamics, although study type (observational (reference category) vs. experimental) was a significant predictor of effect size, with experimental studies producing more strongly negative effect sizes.

Fixed effects	Estimate	SE	*Z*	*p*	Lower CI	Upper CI
Intercept	0.297	0.219	1.361	.174	−0.131	0.725
**Study type (experimental)**	**−0.195**	**0.086**	**−2.272**	**.023**	**−0.364**	**−0.027**
Study method (longitudinal)	0.001	0.067	0.017	.986	−0.131	0.133
Sex (studies including only males)	−0.001	0.024	−0.044	.965	−0.048	0.046
Sample size (> median)	−0.021	0.061	−0.350	.726	−0.141	0.098
Publication year (mean‐centred)	0.003	0.004	0.778	.436	−0.005	0.011
**Random effects**	**Estimate**	***I*** ^**2**^				
Between study variance (Study ID)	0.0002	3.255				
Within study variance (Effect ID)	0	0.553				
Species variance (Species ID)	0.0054	94.355				

*Note*: Study method (cross‐sectional (reference category) vs. longitudinal), sex (studies also including females (reference category), vs. those including only males), sample size (≤ median (reference category) vs. > median), and mean‐centred publication year had no effect. Overall *I* ^2^ was 98.16%. QE_(df = 22)_ = 47.9807, *p* = .0011; QM_(df = 5)_ = 8.1170, *p* = .1499; AIC_c_ = −33.35277. Significant and near‐significant moderators (*p* < .10) are displayed in bold.

**TABLE A5:** The null model, including Study ID, Effect ID, and Species ID as random effects only, shows a non‐significant negative relationship between sexual ornamentation and telomere length or dynamics.

Fixed effects	Estimate	SE	*Z*	*p*	Lower CI	Upper CI
Intercept	−0.085	0.087	−0.967	.333	−0.257	0.087
**Random effects**	**Estimate**	***I*** ^**2**^				
Between study variance (Study ID)	0.0027	3.281				
Within study variance (Effect ID)	0.022	27.085				
Species variance (Species ID)	0.042	51.558				

*Note*: Overall *I* ^2^ was 81.92%. *Q*_(df = 26)_ = 100.2855, *p* < .0001; AIC_c_ = 11.709.

**TABLE A6:** As in the null model, the phylogenetic model shows a non‐significant negative relationship between sexual ornamentation and telomere length or dynamics. Including phylogeny did not improve model fit (ΔAIC_c_ > −2.00).

Fixed effects	Estimate	SE	*Z*	*p*	Lower CI	Upper CI
Intercept	−0.100	0.133	−0.747	.455	−0.361	0.162
**Random effects**	**Estimate**	***I*** ^**2**^				
Between study variance (Study ID)	0	0				
Within study variance (Effect ID)	0.022	26.372				
Species variance (Species ID)	0	0				
Phylogenetic variance	0.046	55.814				

*Note*: Overall *I* ^2^ was 82.19%. *Q*_(df = 26)_ = 100.2855, *p* < .0001; AIC_c_ = 10.326.

**TABLE A7:** The full meta‐regression model shows no relationship between sexual ornamentation and telomere length or dynamics, although study type (observational (reference category) vs. experimental) was a significant predictor of effect size, with experimental studies producing more strongly negative effect sizes.

Fixed effects	Estimate	SE	*Z*	*p*	Lower CI	Upper CI
Intercept	0.682	0.690	0.989	.323	−0.670	2.034
**Study type (experimental)**	**−0.424**	**0.217**	**−1.950**	**.051**	**−0.850**	**0.0021**
Study method (longitudinal)	0.015	0.110	0.131	.896	−0.202	0.230
Temperature regulation (ectotherm)	−0.365	0.355	−1.028	.304	−1.061	0.331
Sex (studies including males only)	0.185	0.124	1.488	.137	−0.059	0.429
Sample size (> median)	−0.097	0.122	−0.791	.429	−0.337	0.143
Publication year (mean‐centred)	−0.013	0.025	−0.529	.597	−0.063	0.036
**Random effects**	**Estimate**	***I*** ^**2**^				
Between study variance (Study ID)	0	0				
Within study variance (Effect ID)	0.016	8.468				
Species variance (Species ID)	0.0024	1.303				
Phylogenetic variance	0.150	81.552				

*Note*: Study method (cross‐sectional (reference category) vs. longitudinal), temperature regulation (endotherm (reference category) vs. ectotherm), sex (studies also including females (reference category) vs. those including only males), sample size (≤ median (reference category) vs. > median), and mean‐centred publication year had no effect. Overall *I* ^2^ was 91.32%. QE_(df = 20)_ = 71.6622, *p* < .0001; QM_(df = 6)_ = 8.2862, *p* = .2179; AIC_c_ = 20.52569. Significant and near‐significant moderators (*p* < .10) are displayed in bold.
